# New insights into inflammatory memory of epidermal stem cells

**DOI:** 10.3389/fimmu.2023.1188559

**Published:** 2023-05-31

**Authors:** Dapeng Cheng, Xiaochen Zhu, Shaochen Yan, Linli Shi, Zhi Liu, Xin Zhou, Xinling Bi

**Affiliations:** ^1^ Department of Dermatology, Changhai Hospital, Naval Medical University, Shanghai, China; ^2^ Department of Dermatology, Affiliated Hospital of Nanjing University of Chinese Medicine, Nanjing, China

**Keywords:** inflammatory memory, epidermal stem cells, hair follicular bulge, epigenetic memory, chromatin accessibility

## Abstract

Inflammatory memory, as one form of innate immune memory, has a wide range of manifestations, and its occurrence is related to cell epigenetic modification or metabolic transformation. When re-encountering similar stimuli, executing cells with inflammatory memory function show enhanced or tolerated inflammatory response. Studies have identified that not only hematopoietic stem cells and fibroblasts have immune memory effects, but also stem cells from various barrier epithelial tissues generate and maintain inflammatory memory. Epidermal stem cells, especially hair follicle stem cells, play an essential role in wound healing, immune-related skin diseases, and skin cancer development. In recent years, it has been found that epidermal stem cells from hair follicle can remember the inflammatory response and implement a more rapid response to subsequent stimuli. This review updates the advances of inflammatory memory and focuses on its mechanisms in epidermal stem cells. We are finally looking forward to further research on inflammatory memory, which will allow for the development of precise strategies to manipulate host responses to infection, injury, and inflammatory skin disease.

## 1 Introduction

Immune memory has long been regarded as one of the critical functions of the adaptive immune system. However, recent studies show that innate immune cells also show adaptive immune functions. When encountering a second insult, a nonspecific enhanced or attenuated reaction could be elicited in innate immunocytes. This phenomenon is termed trained immunity or innate immune memory, whose occurrence is related to epigenetic modification or cell metabolic transformation (1–3). Innate immune memory has a wide range of manifestations. For instance, vaccination and certain infections could induce nonspecific protection against more pathogens via innate immune mechanisms (4). Even plants and invertebrates without adaptive immune systems show immune memory (5). Usually, immune memory will lead to an enhanced immune response to second stimulation. However, low doses of lipopolysaccharide (LPS) could also induce a weaker inflammatory response to external stimuli (6). All these findings support that the innate immune system has adaptive characteristics and innate immune memory of inflammatory processes can be gained during certain immune events and response by multiple cell lineage to various stimuli.

As a broader responsive form of immune memory, inflammatory memory exhibits protective or deleterious responses to the stimuli for the second time. Exploring inflammatory memory will be necessary to understand better the mechanisms of host defense, wound healing, autoimmune-related diseases, aging, and cancer development (7, 8). Previous studies have shown that hematopoietic stem cells (HSCs), fibroblasts, and epithelial stem cells (SCs) from the respiratory and intestinal tract have the function of inflammatory memory (9–11). A recent conceptual advance in the stem cell field is the inflammatory memory of epidermal stem cells (EpdSCs). In 2017, Naik et al. found that the wound healing process sped up after the skin of mice was transiently exposed to imiquimod (IMQ, a topical immune response modifier), which suggests that EpdSCs can gain inflammatory memory to tissue damage. When the skin is injured at the same site, this memory of EpdSCs can contribute to a quicker re-epithelialization process (12). Recently, Levron et al. reported a new wound-distal memory of progenitors derived from SCs in adult mice’s hair follicle junctional zone (13).

This comprehensive review described the definition, mechanisms, and various modalities of inflammatory memory. For instance, HSC, fibroblasts, and epithelial SCs from the respiratory and intestinal tract have all been reported to be involved in memory processes (9, 11, 14). We further focused on the inflammatory memory of EpdSCs and their clinical significance. Highlighted findings in recent years are that certain chromatin regions of EpdSCs remain accessible for up to 6 months after the initial inflammatory stimulation of the epidermis via single-cell sequencing and ATAC-seq analysis. The results showed that epigenetic modifications such as methylation and acetylation occurred in this memory region (15, 16). Overall, inflammatory memory takes part in the pathogenesis of wound healing, chronic immune-related skin diseases, skin aging, and skin tumors.

## 2 Overview of the inflammatory memory

Classically, antigen-specific T or B lymphocytes have immune memory functions. In recent years, inflammatory memory as a form of trained immunity has been fully shown in innate immune cells, which cause modified immune responses to nonspecific second stimulation (17–19). Many cell lineages have been shown to have inflammatory memory functions. For example, hematopoietic cells, fibroblasts, respiratory and intestinal epithelial SCs could independently or cooperatively distribute and store inflammatory memory (9, 11, 20). Theoretically, cells or their progenitors, who persist between the initial immune event and subsequent recall, might preserve inflammatory memory. Especially those long-lived SCs are more likely to be triggered by inflammatory cytokines and acquire inflammatory memory (11, 12, 14). **Table 1** summarizes various inflammatory memory phenomena and mechanisms found so far.

**Table 1:** Diversity of inflammatory memory.

Events of inflammatory memory	Effects of inflammatory memory	Cell types	Mechanisms involved	References
BCG reprograms HSCs in the bone marrow and enhances myelopoiesis	BCG-educated HSCs generate epigenetically modified macrophages that provide significantly better protection against Mtb	HSCs	IFN-related signaling	(21, 22)
Poly (I:C) stimulated human lung fibroblasts	TNFα induced increased release of IL-6	Fibroblasts	Increased secretion of inflammatory mediators such as IL-6 and IL-8	(23, 24)
Allergic disease in people with chronic rhinitis polyps	Th2 cytokines stimulated polyp SCs, which showed increased transcriptional levels and enhanced Wnt/β-catenin pathway	Respiratory epithelial SCs	Sustained activation of genes associated with Th2 cytokines IL-4 and IL-13 in the absence of allergen stimulation	(11, 25)
Low levels of inflammation were induced in mice given a high-fat diet	High-fat diet caused an increase in the stemness of ISCs	ISCs	Activation of the fatty acid sensor PPAR-δ	(26, 27)
Inflammation pretreatment accelerated wound healing of local skin	IMQ-induced psoriasis-like inflammation can alter EpdSCs, resulting in faster repair of subsequent wounds at the same site	Hair follicular bulge SCs	Increased accessibility of the AIM2 gene; synergistic effect of stress responsive transcription factor FOS with JUN and STAT3	(12, 28)
A new wound-distal memory of progenitors derived from SCs in adult mice’s hair follicle junctional zone after an injury	Progenitors in their niche of origin away from the injured site, up to 7 mm display a cell-autonomous transcriptional pre-activated state and enhanced wound repair ability	Progenitors derived from Lrig1^+^ SCs	H2AK119ub-mediated transcriptional de-repression	(13, 29)

HSCs are programmed to produce peripheral blood cellular components. They are pluripotential and can generate diverse mature functional hematopoietic cells. A series of exciting experiments have shown inflammatory memory in HSCs. For example, Kaufmann et al. reported a significant activation of HSCs after vaccination of Mycobacterium tuberculosis (Mtb). HSCs could further pass on this information to macrophage and monocyte progeny, which produce large amounts of inflammatory mediators that kill Mtb more efficiently. On the other hand, Mtb could reprogram HSCs via a type I IFN response, suppressing myelopoiesis and impairing the trained protective immunity against Mtb. The effect may be long-lasting because of chromatin alterations in macrophages from vaccinated mice (21, 22).

Fibroblasts are the most common structural cells in connective tissue and essential immunomodulatory cells. They are essential producers of inflammatory mediators in homeostasis, wound healing, fibrosis and inflammatory diseases (10, 30). Fibroblasts have been demonstrated to function as innate immune cells and maintain a memory, or trained immunity, to repeated inflammatory stimuli (10, 30). The memory is maintained through a variety of mechanisms including DNA methylation, histone modifications and changes in histone abundance, miRNAs, sustained transcription factor and signalling pathway activities, and their metabolic state (23, 24, 30, 31). Fibroblasts are becoming essential research subjects in chronic inflammatory skin diseases such as psoriasis, scleroderma, and vitiligo (32–34).

The development, update, and homeostasis of the airway and lung monolayer epithelia are regulated by pluripotent epithelial progenitors or SCs (35–37). These cells are ideal candidates for recalling immunological events because terminally differentiated cells constantly shed off and cannot live long enough (38). Recently, different teams have carried out many inflammatory memory studies of respiratory epithelial SCs in human allergic diseases (11, 38, 39). The results revealed striking changes in epithelial cell diversity and subtypes via scRNA-seq of chronic rhinosinusitis samples (11). In addition, when stimulated by Th2 cytokines IL-4 and IL-13, cultured polyp SCs revealed an enhanced transcriptional response and activation of Wnt/β-catenin pathways compared with non-polyp tissue, which showed that polyp SCs have specific memories of allergic reactions they have experienced *in vivo*. The efficacy of dupilumab, a fully human monoclonal antibody that inhibits the signaling of Th2 cytokines IL-4 and IL-13, in the treatment of patients with chronic nasal polyps further demonstrates this inflammatory memory (11, 25, 40). Some studies suggest that respiratory epithelial SCs may contribute to the persistence of human allergic diseases by acting as a repository of allergic memory (11). The memory mechanism may be related to internal, external, and epigenetic factors that lock basal polyp cells in an uncommitted state (11, 25). However, the specific mechanism needs to be further studied.

The intestinal stem cells (ISCs) include actively cycling Lgr5+ columnar cells and slower cycling Bmi1+ cells (41–44). In 2016, Beyaz et al. showed that a high-fat diet augments the numbers and function of Lgr5+ ISCs of the mammalian intestine by inducing a robust Peroxisome proliferator-activated receptor delta (PPAR-δ) signature in ISCs and progenitor cells (45). In 2017, Unnikrishnan et al. found that dietary restriction can induce changes in gene expression in mice, which persist even when a dietary restriction is discontinued. DNA methylation of the Nts1 gene in ISCs may play a role in this memory effect (26). In 2021, Mana et al. further demonstrated that a high-fat diet enhances intestinal stemness and tumorigenicity (46). Recently, Reddy et al. found that inflammation from gastro-intestinal acute graft-versus-host disease leaves a memory of its effects on ISCs that persist and are likely to affect their sensitivity to adapt to future stress or challenges. They found that Lgr5+ ISCs undergo metabolic changes that lead to the accumulation of succinate, which reprograms its epigenome (27). Exploring the inflammatory memory of ISCs is essential for maintaining intestinal homeostasis and preventing and treating intestinal diseases.

## 3 Current research on EpdSCs

The epidermis is the critical barrier structure of the body. It contains at least three distinct stem cell populations. They are situated within the hair follicle bulge, the junctional zone, and the basal layer of the interfollicular epidermis (IFE) (47, 48). The identification and isolation of SCs are usually made by functional and lineage tracking analysis. EpdSCs have been purified based on the expression of some unique surface markers, including integrin, keratin (K), P63, and CD34. When labeled by administration of nucleotide analogs such as [3H] thymidine or bromodeoxyuridine (BrdU), the retention process will remain for a prolonged time. It is well known that EpdSCs divide asymmetrically, giving rise to a transit-amplifying (TA) cell and one parent stem cell. The rapidly cycling TA cells gradually lose the label, undergoing further differentiation (49). However, SCs that divide less frequently keep the label and are called label-retaining cells (LRC). This division pattern provides and sustains many SCs that update the tissue continuously. EpdSCs maintain skin homeostasis and hair regeneration, especially in the epidermal repair after injury. The immune memory function of EpdSCs has become an important research field in recent years (17, 47, 50, 51).

### 3.1 Hair follicular bulge SCs

**Figure 1:**
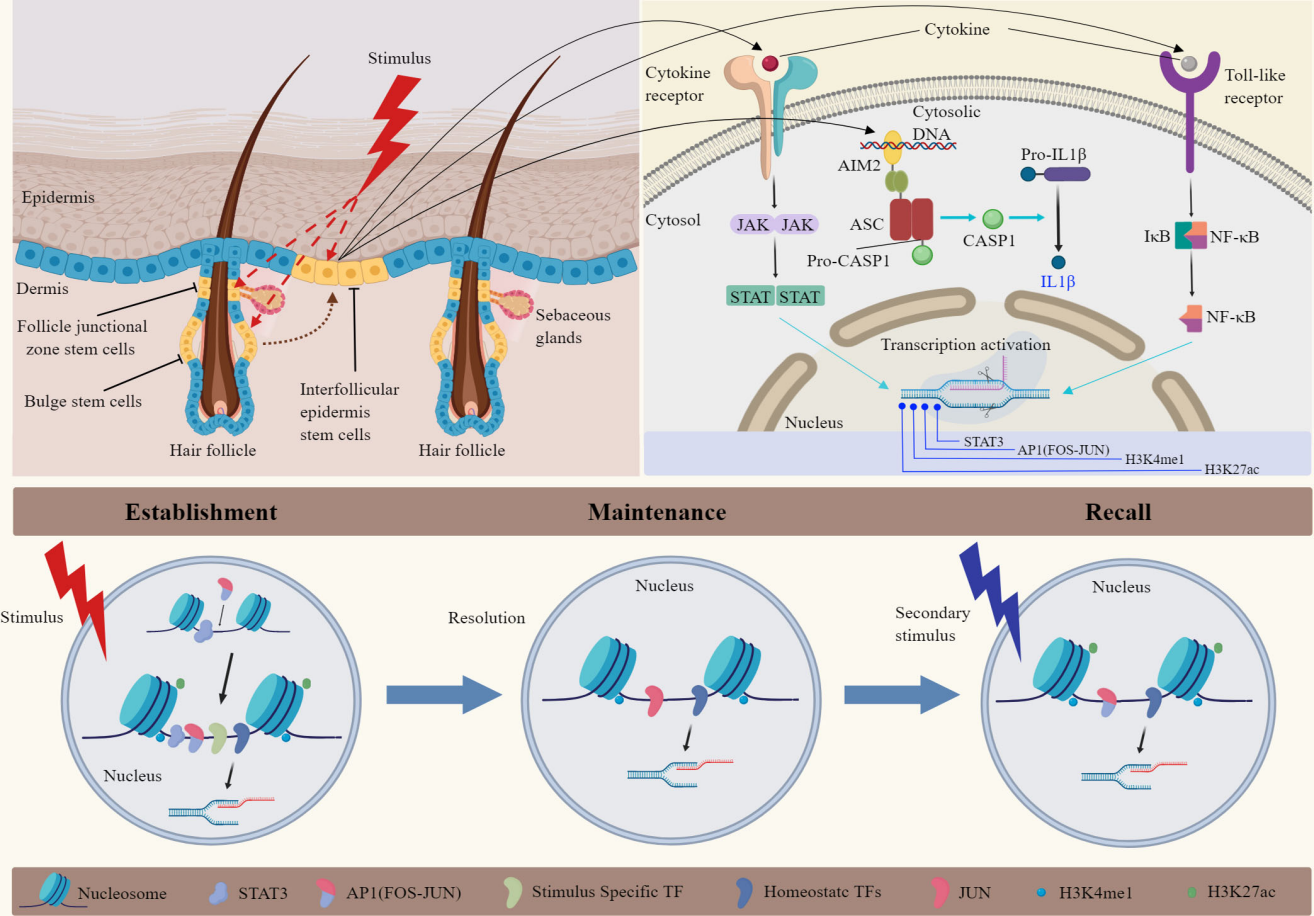
Mechanisms of inflammatory memory in epidermal stem cells. Hair follicle SCs play significant roles in skin homeostasis and injury repair. Inflammatory memory could be established, maintained, and recalled by epidermal SCs following various stimuli. The mechanism is related to chromosomal accessibility in epidermal SCs. Single-cell sequencing and ATAC-seq analysis showed that the primary stimulation activates AIM2, transcription factors (STATs, NF-κB), and remodel chromatin at inflammatory stress response genes. After inflammation remission, epidermal SCs maintain accessibility and histone modification in specific chromatin loci. When the skin was injured for the second time, FOS was rapidly recruited to memory domains, leading to the re-activation of AP1-dependent transcription. Important epigenetic markers such as H3K27ac and H3K4me1 contributed to chromatin accessibility in this process. JAK, Janus Kinase; STAT, signal transducer and activator of transcription; AIM2, absent in melanoma 2; CASP1, caspase-1; AP1, activator protein-1; NF-κB, nuclear transcription factor-κB; TF, transcription factors.

The bulge of the hair follicle, as part of the outer root sheath, is a repository of EpdSCs. Analysis of dynamic lineage progression and transcriptomic changes in mouse hair follicle epithelium revealed that bulge SCs originate from the periphery of the placode basal layer. They could migrate to the hair follicle matrix, the sebaceous gland, and the basal layer of the IFE to produce progenitors that differentiate into cells of hair, gland, or epidermis (52, 53). The upper segment of the hair follicle is permanent and comprises the infundibulum and isthmus. The lower part is transient and goes through growth, resorption, and rest phases, often called the anagen, catagen, and telogen stages. Bulge SCs do not contribute to maintaining the IFE under typical homeostasis situations, but can rapidly migrate upward and repair the epidermis after skin injury (48, 54) (**Figure 1**). With burns, the regenerative function of the hair bulge is highlighted. Superficial burns with intact appendage structures allow rapid healing and regeneration of epidermal appendages. When severe burns involve the hair bulge, the skin can regenerate without leaving behind damage to adnexal structures and scar formation.

Various markers consistent with “stemness” were used to identify SCs in the epidermis. Hair follicle bulge SCs have been purified by fluorescence-activated cell sorting based on the expression of surface markers, including K15, CD34, Lgr5, and α6 integrin (55). Most studies isolate and sort EpdSCs from rodent animal skin. In humans, although neonatal foreskin is the typical source of normal keratinocytes, SCs in the hair follicle bulge can often be obtained from hairy skin, such as the human scalp, with positive sorting markers K15, β1 integrin, and CD200 (56). In addition, the supply of human hair follicles for investigative purposes is limited in most conditions. The role of hair follicle bulge SCs in inflammatory memory is derived from mice’s scRNA-seq and ATAC-seq analysis in the latest studies (57–59).

### 3.2 SCs in the hair follicle junctional zone

The EGF receptor antagonist leucine-rich repeats and immunoglobulin-like domains 1 (Lrig1) have been identified as one marker of human EpdSCs (60). In 2009, Jensen et al. found that Lrig1 expression specifies a unique population of EpdSCs in the mouse epidermis, located in the hair follicle junctional zone adjacent to the sebaceous glands and infundibulum. As bulge SCs did in epidermal reconstitution experiments, these cells could contribute to all epidermal lineages (61). Then, in 2013, Page et al. demonstrated that Lrig1^+^ cells are highly proliferative EpdSCs and can maintain the upper pilosebaceous unit, containing the infundibulum and sebaceous gland as independent compartments, but contribute to neither the hair follicle nor the IFE by Long-term clonal analysis (29). They also found that stem cell progeny from multiple compartments gain lineage plasticity and permanently contribute to regenerating tissue by analyzing three-dimensional reconstructions of epidermal-tail whole mounts after wounding (29).

### 3.3 Interfollicular epidermis SCs

The IFE SCs form a watertight barrier and play a significant role during skin homeostasis. The detection of interfollicular LRC confirmed the existence of IFE SCs in the human and mouse IFE basal layer (62). Compared with hair follicle SCs, human IFE SCs showed high expression of β1 integrin, α6 integrin, and delta1 and low expression of CD71 (63). In addition, as one of the identified markers of human IFE SCs, p63 is an essential transcription factor for epidermal development and homeostasis. It controls the fate of keratinocytes by regulating the balance of stemness, differentiation, and senescence (64, 65). Recently, human IFE SCs have been reported to be identified by the expression of a novel combination of p63 and histone deacetylase1 (HDAC1) (66). Although there is no evidence to confirm the role of IFE SCs in inflammatory memory, the recurrence of inflammatory skin diseases such as psoriasis and atopic dermatitis is more common in hairless skin, mechanism of local recurrence needs to be further studied.

IFE SCs contribution to epidermal homeostasis is supported by slow-cycling cells in the basal layer and the clonal regeneration of the mouse IFE (67, 68). However human IFE has some unique characteristics from the mouse. The human epidermis has more layers of keratinocytes in most body sites, projecting into the dermis as rete ridges and separated by dermal papillae. Identifying and isolating human IFE SCs is difficult due to the lack of specific stem cell markers. Recently, it has been proposed that human IFE may be maintained by progenitor cells behaving similarly to those in the mouse, allowing the clusters of SCs to remain quiescent (68). In addition, IFE SCs are believed to play a significant role in dermal homeostasis in hairy skin (69). However, human skin includes some hairless skin, such as palms, soles, glans, and nipples, and the role of IFE SCs in these areas needs further investigation.

## 4 Inflammatory memory of EpdSCs

The inflammatory memory of EpdSCs enables the body to produce enhanced or eased responses when it receives external stimuli a second time. Repeated inflammatory memory may also be involved in the recurrence or induction of immune-mediated skin diseases, skin aging, and the development of skin cancers (70). How to intervene or block the harmful inflammatory memory of SCs has a significant theoretical and research value. More and more research on the occurrence and mechanism of inflammatory memory in EpdSCs has been reported in recent years.

### 4.1 Remember and respond to various inflammatory stimuli by EpdSCs

EpdSCs of the skin sense and respond to various inflammatory stimuli, including bacteria, viruses, injuries, and other environmental factors, to maintain and repair tissues in health and disease. It was previously acknowledged that only some immune cells can gain memory to resist exogenous stimuli. However, in 2017, Professor Fuchs and her team reported that when inflammation or trauma occurs to the skin, EpdSCs in mice perceive stimuli, proliferate, and differentiate to replace the damaged epidermal cells. Even if the stimuli disappeared, some EpdSCs maintained a post-inflammatory situation for a long time, contributing to a sped-up wound-healing process (12). It is well known that most epidermal cells transition from the basal layer to the stratum corneum and eventually fall off. Keratinocytes do not stay long enough to maintain this “memory,” while EpdSCs divide slowly *in vivo* and have slow cell cycle properties. Therefore, the sensitivity of the epidermis to secondary stimuli may be related to the memory of inflammation in EpdSCs. Finally, based on their mouse experiments, the hypothesis was confirmed (12, 15). In another experiment, Zhang et al. gave the mice IMQ again at the same site after the first inflammatory response had subsided and found that the second inflammatory response appeared faster and more severe, which suggests the inflammatory memory after skin inflammation (71).

Recently, Levron et al. reported a new wound-distal memory of progenitors in adult mice. They found that after a first injury, Lrig1^+^ SCs give rise to long-term wound-memory progenitors residing in their niche of origin away from the injured site, up to 7 mm. These newly identified wound-distal memory cells display a cell-autonomous transcriptional pre-activated state, enhancing wound repair ability. When the damage has been resolved, and new homeostasis has been re-set, Lrig1^+^ SCs derived cells remain transcriptionally pre-activated (13).

### 4.2 Mechanisms of inflammatory memory in EpdSCs

The process of inflammatory memory is related to tissue adaptation to environmental exposures during homeostasis and disease conditions. Theoretically, this process might occur on any cell type, especially skin cells and various barrier epithelial cells. It will be essential to determine the distribution and interaction of different cell lineages and how they are shaped by host and environmental factors (38). Recent studies have shown that EpdSCs can record inflammatory events by changing their chromatin landscape and function (1, 7).

The localization of inflammatory memory is mainly concentrated in the chromatin of EpdSCs (**Figure 1**). Epigenetic or metabolic changes enhance the skin sensitivity and tissue repair capacity to subsequent encounters (8). The inflammatory response makes chromatin remodeling and activates inflammation-related transcription factors (14). Upon resolution, EpdSCs retain inflammation-induced chromatin accessibility. These chromatin domains contain inflammation-sensing regulatory elements and genes associated with enhancers (72). These elements are also related to genes that encode skin barrier restoration proteins. After a subsequent skin injury stimulus, SCs show enhanced transcriptional response and promote wound repair. Further investigation exploring the mechanisms of inflammatory memory showed no contributions of skin-resident T cells or macrophages. The inflammasome, IL-1β, and epigenetic reprogramming of SCs have been involved. Significantly, the absence of melanoma 2 (AIM2)/caspase-1/IL-1β axis seemed essential for memory recall. AIM2 is the crucial memory mediator and inflammasome activator, and it augments IL-1β to promote the regenerative process after injury (14). EpdSCs express a variety of cytokine and pattern recognition receptors that could sense damage-associated molecular patterns (DAMPs), pathogen-associated molecular patterns (PAMPs), and cytokine signals (73). In addition, the inflammatory memory state in EpdSCs could persist for at least 180 days, showing the durability of the response.

Some researchers want to know how epigenetic memory works and how chromatin information can be transmitted to progeny after SCs divide. They found that inflammation-associated transcription factors, such as STATs and NF-κB, are activated rapidly through post-translational mechanisms (**Figure 1**). As the essential element for innate immune responses to infection or tissue damage, NF-κB execute function via conventional TNF-α/TNFR/NF-κB signal pathway in the skin. Some studies reported that specific chromatin sites containing some sequence of inflammation-activated TFs remain open status. Although the molecular details underlying inflammatory memory are obscure, DNA or histone modifications play a significant role in maintaining inflammatory memory (74). EpdSCs infrequently divide, which may permit more epigenetic modification to be sustained. However, as the primary form of epigenetic modification, histone modifications are relatively unstable in their inheritance compared with DNA methylation. So, more exciting mechanisms will be explored to understand the inflammatory memory phenomenon better.

The latest mechanisms of inflammatory memory related to epigenetic inheritance are detailed below (**Figure 1**). After an initial inflammatory or microbial trigger, SCs change histones and chromatin accessibility to activate the transcription of inflammatory, antimicrobic, and stress-related genes. Most of these genes could return to their baseline epigenetic status after stopping stimulus, but restoring H3K4me1 modification at enhancers or H3K4me3 modification at proximal promoters is slow (28). Associated genes of accessible chromatin permit the rapid recruitment of RNA polymerase II and transcriptional activation upon a secondary trigger (75). Levron et al. found that the long-range priming relies on H2AK119ub-mediated transcriptional de-repression in distal memory cells derived from Lrig1^+^ SCs (13). However, whether histone modifications are enough to maintain memory domains in an open status without inflammation and what kind of secondary stimuli could trigger the memory need to be explored.

Besides the histone modifications of enhancer H3K4me1 and promoter H3K4me3 in the open chromatin region mentioned above, other epigenetic modifications include histone-change enzymes and nucleosomes that could bind to multiple transcription factors. Researchers focused on inflammation-sensing transcription factors that are activated because of cytokine or microbial exposure. For example, JAK/STAT signaling is essential for the initial response, establishing inflammatory memory (76). In addition, many members of the Stat family are rapidly induced by various cytokines in the recall stage. Recently, Larsen’s study showed that establishing memory requires stimulus-specific factor STATs to be activated and open the memory domains and the general stress-responsive transcription factors of the AP-1 family, including c-JUN and c-FOS, to remodel and open the chromatin. They found pre-existing inflammation-independent transcription factors bind to inflammatory memory loci and preserve accessibility long after the inflammation; stress-responsive transcription factors are no longer present. Above all, FOS-associated AP-1 factors are more important for establishing and recalling inflammatory memory from analyzing existing databases in human and mouse cells (16). The present studies show that EpdSCs establish, maintain, and recall immune memory through various sensors, then alter their responses and function. However, it remains to be explored how inflammatory memory maintained by EpdSCs influences stress-induced ligands, which further interact with T lymphocytes or inflammatory mediators in the local niche (77).

### 4.3 Clinical significance of inflammatory memory in EpdSCs

Recent discoveries revealed that epidermal stem cell memory might participate in various pathophysiological conditions. For example, inflammatory memory could change the skin wound healing process. However, memories can also be maladaptive, leading to chronic immune-related skin disorders and skin cancer development. Enhancing the beneficial memory effect and reducing harmful inflammatory memory will be of great value in preventing and treating related skin diseases.

#### 4.3.1 Inflammatory memory in wound healing

The effect of inflammatory memory on skin wound healing is an important research field for training immunity. Levy et al. reported that follicular SCs contribute to the resurfacing of the wound and could remain resident in the basal layer of the epidermis months later (78). The division of EpdSCs, affected by various inflammatory niches, could repair the wound. The wound-healing process is usually divided into at least three stages: coagulation, inflammation, and repair. Many cytokines and chemokines play a significant role in tissue regeneration during the wound inflammation phase. The pioneering study of Naik on the inflammatory memory of EpdSCs is carried out in psoriasis-like mice induced by IMQ. They found that skin wounds at the inflammation resolution site heal faster than control mice independently of immune cells, such as resident T cells and macrophages. Chromosomal accessibility in EpdSCs was identified by high-throughput sequencing, and IMQ exposure increased the accessibility of the AIM2 gene, which is related to inflammation and hyper-proliferation in EpdSCs. AIM2-deficient mice lost the ability to recollect inflammation and failed to speed up wound repair in previously inflamed skin. When inducing the expression of epidermal AIM2, wound repair was enhanced even without pre-challenge with IMQ (12). There are also some clinical phenomena associated with inflammatory memory. For example, a surgical incision or punch injury can induce new lesions on the normal skin of psoriasis patients, which is termed the Koebner phenomenon (79, 80). Conversely, the acceleration phenomenon observed in animal wound healing models through inflammatory memory still lacks enough clinical work support.

#### 4.3.2 Inflammatory memory in immune-related skin diseases

Psoriasis and atopic dermatitis are common chronic inflammatory skin diseases. Inflammatory memory may play an essential role in the recurrence of these diseases. In clinical work, recurrence of psoriasis lesions often occurs in friction areas such as the back, elbows, and anterior tibia of lower extremities. Despite psoriatic lesions subsiding and the skin with an almost normal appearance after therapeutic intervention, transcriptional profiling of resolved skin with persistent differential expression of disease-related genes distinguishes it from uninvolved healthy skin (81). It is usually believed that tissue-resident memory T lymphocytes have a role in the recurrence of psoriasis (82). However, in the mouse model of psoriasis-like inflammation, the memory function of EpdSCs rather than tissue-resident T cells was confirmed.

Transcriptome and ATAC-seq analysis of psoriatic and normal skin tissues showed that AP-1-mediated genes might regulate the histopathological changes of psoriatic lesions (83). In an IMQ-induced psoriasis-like mouse model, the chromatin alteration of EpdSCs was found and could persist for a long time in the skin of inflammation resolution. The same mouse model showed that over 1,000 DNA regions in EpdSCs gained accessibility at the peak of skin inflammation. Essential mechanisms are that stress-responsive transcription factor FOS cooperates with JUN, signal transducer, and activator of transcription 3 (STAT3). After resolving the inflammatory response, STAT3 and FOS were released from the memory domains. However, JUN, ATF3, and p63 remained on memory domains, which could quickly maintain the chromatin open at the memory domains and facilitate FOS recruitment and gene reactivation on secondary challenges such as wounding and infections (16). These results show that EpdSCs could gain long-term epigenetic memory during psoriasis. Chromatin accessibility increases in psoriasis, and two epigenetic markers are vital contributors (32, 84). One is histone three lysine twenty-seven acetylation (H3K27ac) at distal enhancers could make the acetyl group neutralize the positive charge of histone three lysine twenty-seven (H3K27) and lead to weaker interactions between histone and DNA. Another is histone three lysine four trimethylation (H3K4me3), which has nucleosome remodeling roles at the promoters of stimulated genes. The inflammatory memory could make EpdSCs more vigorous in response to a broad range of subsequent stressors, potentially contributing to the recurrence of psoriasis.

#### 4.3.3 Inflammatory memory in skin tumors

Abnormal proliferation and differentiation of EpdSCs lead to the occurrence of squamous cell carcinoma (SCC) and basal cell carcinoma (BCC). It was reported that epidermal cells positively stained with LGR5, one of the crucial markers of SCs in the epidermis, might be BCC’s cell origin (85), and the LGR5-positive tumor population is closely related to basal cell carcinoma relapse (86). Chronic inflammation plays a significant role in the occurrence and development of SCC and BCC because of various physicochemical factors. For example, skin SCC is prone to occur based on discoid lupus erythematosus, lichen planus, and chronic cheilitis.

Multiple studies found that repeat inflammatory stimuli can induce inflammatory memory in EpdSCs, resulting in related gene mutations and keratinocyte abnormal proliferation and differentiation (12, 13, 87). Chickens infected with the Rous sarcoma virus usually develop tumors along the wound site after an injury (88). In mice, inflammatory memory of EpdSCs after subsequent tissue damage increased the susceptibility to cancer (12, 13). In addition, inflammatory training of tumorigenic cells can confer enhanced cancer susceptibility in human (89).

Baksh et al. highlighted that oncogenic EpdSCs are serine auxotrophs whose growth and self-renewal require abundant exogenous serine. EpdSCs activate *de novo* serine synthesis when extracellular serine is limited, removing the repressive histone modification H3K27me3 and activating differentiation programs that promote SCC (90). The literature has reported that H3K27me3 is related to β-glucan-trained immunity (91); thus, the mechanism of the relationship between EpdSCs inflammatory memory and SCC development needs to be further studied.

## 5 Conclusions and future perspectives

Inflammatory memory in EpdSCs is one kind of trained immunity, like in the innate immune system’s monocytes, macrophages, and natural killer cells. However, SCs are characteristic of multipotency and could transfer the epigenetic modification to next-generation cells, resulting in the training of the entire tissue (92). Besides hair follicle bulge SCs in mice, there still needs to be more in-depth research on whether the SCs of sebaceous glands and sweat glands involve the formation of inflammatory memory. Although some progress has been made in establishing and maintaining inflammatory memory in EpdSCs, the time and intensity of its occurrence need to be further studied between pathophysiological conditions.

EpdSCs collect information about various stimuli and remember and respond to subsequent exposures. The responses are changed to reduce damage, repair the tissue, and maintain homeostasis. However, the inappropriate intensity of an active immune response will cause chronic inflammation. A similar mechanism is involved in various inflammation-related diseases, such as chronic wounds, psoriasis, atopic dermatitis, and skin cancer. Although the memory domains could be explained by chromatin accessibility, the critical question is, what incentives can stimulate specific chromatin regions to form long-term epigenetic alteration? Are other epigenetic markers contributing to chromatin accessibility besides H3K27ac, H3K27me3, and H3K4me1 (93)? Is there heterogeneity in the same or different lineage SCs? Can we artificially activate memory loci to regulate the interaction between memory SCs and the local immune niche? A more comprehensive and in-depth study on the molecular mechanisms underlying immune memory formation in EpdSCs is necessary. Modulating the epigenetic reprogramming of inflammatory memory may offer novel therapeutic strategies for wound healing, immune-related skin diseases, and skin tumors.

## Author contributions

DC and XCZ contributed to the conception and design of this review. SY, LS, and ZL contributed to the survey and visualization of the review. DC, XCZ, SY, LS, and ZL wrote sections of the manuscript. XB and XZ contributed to the review and editing of the manuscript. All authors contributed to the article and approved the submitted version.
